# Reproductive tradeoff limits the predatory efficiency of female Arizona Bark Scorpions (*Centruroides sculpturatus*)

**DOI:** 10.1186/1471-2148-13-197

**Published:** 2013-09-14

**Authors:** Michael M Webber, Javier A Rodríguez-Robles

**Affiliations:** 1 School of Life Sciences, University of Nevada, Las Vegas, 4505 S. Maryland Parkway, Las Vegas, Nevada 89154-4004, USA

**Keywords:** Cost of reproduction, Foraging, Life history tradeoff, Maternal care, Predatory efficiency

## Abstract

**Background:**

Life history tradeoffs may result from temporal and physiological constraints intrinsic to an organism. When faced with limited time and energy, compromises occur and these resources are allocated among essential activities, such as body growth, maintenance, foraging, mating, and offspring care. We investigated potential tradeoffs that may occur between reproductive activities and feeding performance in female Arizona Bark Scorpions (*Centruroides sculpturatus*) by comparing the time taken to capture prey between non-reproductive and reproductive females (gravid females and females exhibiting maternal care, i.e. carrying offspring on their backs).

**Results:**

Gravid females were as efficient at catching prey as non-gravid females. To control for variation in the duration of the maternal care period, we removed all offspring from all post-parturient females after 5 days. Brooding females and females 24 hours following offspring removal (FOR) did not successfully capture prey within the 900-second trial period. Twenty-eight days FOR, females caught prey faster than females displaying maternal care and females 24 hours FOR, but were not as efficient at catching prey as non-gravid and gravid females. When pursuing prey, *C. sculpturatus* exhibiting maternal care used an active foraging strategy more frequently than non-gravid, gravid, and females 28 days FOR. In contrast, non-gravid, gravid, and females 28 days FOR used active and ambush foraging with similar frequency.

**Conclusions:**

Our data suggest that reproduction does not significantly reduce the predatory efficiency of gravid *C. sculpturatus*, and that these females can cope with increasing body mass and the physiological costs of gestation. However, the observation that brooding females and females 24 hours FOR did not catch prey within the trial period indicates that maternal care significantly reduces predatory efficiency in these scorpions. Females 28 days FOR were still not as efficient at catching prey as non-gravid and gravid females, suggesting that reproductive costs extend for at least 4 weeks after the end of the maternal care period. Preferential use of an active foraging strategy by brooding females may increase prey encounter rates, allowing the scorpions to more rapidly replenish energy reserves depleted during reproduction. However, active foraging may be energetically costly and increase predation risk for brooding females. Our findings regarding antagonistic interactions between reproduction and feeding in female *C. sculpturatus* demonstrate the pervasive nature of reproductive costs for viviparous females, and may provide insight on factors that influence the diversity of reproductive strategies observed in nature.

## Background

Life history theory posits that there are high costs associated with reproduction [1-4], especially for viviparous species [5-7]. The internal development of offspring and the allocation of nutrients to offspring during gestation can impose substantial metabolic costs on viviparous females [8,9]. To reduce high reproductive costs, physiological and behavioral tradeoffs may occur [10], resulting in alterations in the activity patterns of females during a reproductive bout. Additionally, in species that exhibit maternal care, brooding behaviors by females can be demanding activities [11-13] that may extend reproductive costs after parturition, and may potentially conflict with other tasks essential for survival, such as resource acquisition.

Studies examining reproductive costs for females have shown that decreases in locomotor performance and alterations in defensive strategies may occur as a result of increasing body mass during gestation [1,6,14]. Reproductive costs that result in decreased locomotor ability in gravid females may also hinder movements as they pertain to foraging and prey capture. Numerous reports have investigated the influence of resource availability on the reproductive investments made by females (i.e. reproductive frequency, offspring number, and offspring size; [15-18]), but few studies have addressed the potential antagonistic effects of reproduction (i.e. gestation and maternal care) on the foraging and feeding behaviors of females. Herein we investigated how reproductive status may influence trophic resource acquisition in female Arizona Bark Scorpions (*Centruroides sculpturatus*, Ewing 1928 [=*Centruroides exilicauda*, Wood 1863 of some authors]), a species that inhabits rocky hillsides, outcrops, and riparian habitats in southwestern North America [19]. *Centruroides sculpturatus* feed on a variety of invertebrates (e.g. spiders, cockroaches, centipedes [20]), and once prey is captured with the chelae (pincers), *C. sculpturatus* envenomate it via the aculeus (stinger) on the end of the metasoma (tail). Occasionally, small or non-struggling prey items are subdued and consumed without envenomation [19]. Female *C. sculpturatus* are viviparous [21], and following parturition exhibit maternal care by carrying offspring on their backs. Offspring later disperse following their first cycles of ecdysis (molting).

As previously stated, increases in body mass and nutrient allocation to offspring during gestation can result in decreased locomotor performance in reproductive females [1,6,14]. This decrease in locomotor ability may also negatively impact a female’s ability to capture prey. Accordingly, we predicted that reproductive female *C. sculpturatus* (gravid females and females providing maternal care) exhibit a decrease in predatory efficiency, compared to non-reproductive females, and thus take longer to capture prey than non-reproductive females and females following the maternal care period. In addition, to compensate for decreased locomotor performance, reproductive female *C. sculpturatus* may alter their foraging strategy. *Centruroides sculpturatus* exhibit both active and ambush (sit-and-wait) foraging behaviors [21]. Active foraging involves relatively frequent movements by predators, and often incurs greater locomotor costs, compared to ambush foraging [22]. The frequent movements made by active foragers can expose them to a greater predation risk, compared to ambush foragers [23]. Reproductive female *C. sculpturatus* may attempt to reduce the energetic costs of foraging and decrease their predation risk by changing their foraging behavior. Therefore, we also predicted that reproductive female *C. sculpturatus* employ an ambush foraging strategy more frequently than non-reproductive females and post-parturient females.

## Methods

We collected 65 mature female *C. sculpturatus* from the outskirts of Quartzsite (33° 38′ 9″ N, 114° 18′ 15″ W), La Paz County, southwestern Arizona, USA. We weighed and housed all individuals in separate plastic containers (15 × 9 cm) lined with a gravel substrate and maintained at 24.0° ± 5.0°C.all scorpions 24 hours after capture by placing a prey item (Common House Cricket, *Acheta domesticus*) within their enclosures, and allowed the arachnids to acclimate for 14 days prior to the start of the feeding trials. Of the 65 females, 22 were non-gravid and 43 were gravid. To obtain females exhibiting maternal care, 20 of the gravid females were maintained separately until parturition. Female scorpions exhibit maternal care for variable lengths of time ([24]; M. M. Webber, unpublished data), and to control for this variation we removed all offspring from all post-parturient females after 5 days.

We began each feeding trial by introducing a Common House Cricket approximately one-third of the body mass of the scorpion on the opposite end of the container where the scorpion was housed. Scorpions are nocturnal predators, and therefore we conducted all feeding trials under low light conditions, and filmed them using a Sony DCR VX2100 Digital Video Camera Recorder. We measured prey capture time from the time of prey recognition (i.e. alert stance, orientation towards prey, grasp attempt, cheliceral activity [25]) until prey ingestion began. To assess potential changes in predatory efficiency during the later stages of a reproductive bout, we measured anew the prey capture time of females exhibiting maternal care at 24 hours and 28 days following offspring removal (FOR). Each trial lasted a maximum of 900 seconds. Logistical reasons often suggest that a particular trial or study ends at a prespecified time point (cut-off time). The time to the event of interest is known precisely for those subjects that present the desired event before that time point. For the remaining subjects, the time to the event of interest is greater than the observation time, or the event never occurs. This is referred to as administrative censoring, and the incomplete data are called right-censored [26]. Accordingly, prey capture time for females that failed to capture prey within the 900-second trial period was right-censored [27].

We compared the time taken to capture prey among five reproductive classes (non-gravid, gravid, females exhibiting maternal care, and females 24 hours and 28 days FOR) using the non-parametric Kaplan-Meier Failure Time Analysis. This method allowed us to examine the probability of prey capture over time, while accounting for the right-censored data in the study. To avoid pseudoreplication, we only compared prey-handling times among independent groups: (i) non-gravid, gravid, and females exhibiting maternal care; (ii) non-gravid, gravid, and females 24 hours FOR; and (iii) non-gravid, gravid, and females 28 days FOR. We performed pairwise comparisons of prey capture time among reproductive groups using the Mantel-Cox Test (Log Rank Test). We used a Cox-Proportional Hazards Model to examine the potential influence of body size (carapace length × width, mm^2^) and chela size (length × width, mm^2^) on prey capture time of females in each reproductive group. This analysis enabled us to examine the potential effect of covariates on the hazard rates of prey capture among females. In the context of this study, hazard rate refers to the rate at which individuals successfully captured prey at time *t*[28]. Prey capture time was right-censored for females that failed to capture prey within the 900-second trial period, and thus we excluded these individuals from the Kaplan-Meier Failure Time Analysis and the Cox-Proportional Hazards Analysis.

Whenever possible, we recorded the foraging strategy used by each individual, and scored it as either “active searching” (when females pursued the crickets) or “ambush predation” (when females remained stationary until prey capture). We compared the frequency with which females used each foraging behavior using binomial tests (G-tests). We excluded from this analysis trials in which the foraging strategy used by a female was unclear, and in which prey behavior circumvented the use of a given foraging behavior by females (i.e. when the cricket approached the scorpion immediately after being introduced into the enclosure). All statistical analyses were performed using SPSS (SPSS 20 Inc., Chicago, IL, USA). Values reported are means ± 1 SD, and all *P* values are two-tailed.

## Results

Non-gravid and gravid females had similar success rates of prey capture (G = 0.001, *df* = 1, *P* = 0.97; Table 1). Both groups caught prey more frequently than females exhibiting maternal care (non-gravid females vs. females exhibiting maternal care, G = 50.1, *df* = 1, *P* < 0.001; gravid females vs. females exhibiting maternal care, G = 51.4, *df* = 1, *P* < 0.001), and than females 24 hours FOR (non-gravid females vs. females 24 hours FOR, G = 45.7, *df* = 1, *P* < 0.001; gravid females vs. females 24 hours FOR, G = 46.8, *df* = 1, *P* < 0.001). Non-gravid females were twice as likely to capture prey as females 28 days FOR (G = 6.1, *df* = 1, *P* = 0.014; Table 2). Similarly, gravid females were 1.8 times more likely to capture prey than females 28 days FOR (G = 6.3, *df* = 1, *P* = 0.012; Table 2). Females 28 days FOR caught prey more frequently than females exhibiting maternal care (G = 21.0, *df* = 1, *P* < 0.001) and females 24 hours FOR (G = 19.1, *df* = 1, *P* < 0.001).

**Table 1:** **Frequency of prey capture in female *Centruroides sculpturatus***

**Reproductive status**	**Success**	**Failure**
	**frequency (%)**	**frequency (%)**
Non-gravid females	21 (95.5%)	1 (4.5%)
Gravid females	22 (95.7%)	1 (4.3%)
Females exhibiting maternal care	0 (0%)	20 (100%)
Females 24 hours following offspring removal	0 (0%)	17 (100%)
Females 28 days following offspring removal	9 (64.3%)	5 (35.7%)

Global Likelihood Ratio, G = 97.8, *df* = 4, *P* < 0.001.

**Table 2:** **The effect of reproductive status and body size on prey capture time among non-gravid and gravid female *Centruroides sculpturatus*, and females 28 days following offspring removal**

**Covariate**	**Wald**	**Exp (β)**	***df***	***P***
**Non-gravid females (n = 22) vs. Gravid females (n = 23)**				
Reproductive status	0.351	1.20	1	0.554
Body size (carapace length x width, mm^2^)	0.493	0.960	1	0.483
Chela size (length x width, mm^2^)	2.136	1.168	1	0.144
**Non-gravid females (n = 22) vs. Females 28 days following offspring removal (n = 14)**				
Reproductive status	6.649	2.951	1	0.010
Body size (carapace length x width, mm^2^)	0.023	1.009	1	0.880
Chela size (length x width, mm^2^)	0.808	1.111	1	0.369
**Gravid females (n = 23) vs. Females 28 days following offspring removal (n = 14)**				
Reproductive status	5.834	2.813	1	0.016
Body size (carapace length x width, mm^2^)	2.807	0.893	1	0.094
Chela size (length x width, mm^2^)	4.460	1.331	1	0.035

Cox-Proportional Hazards Model.

**Figure 1:**
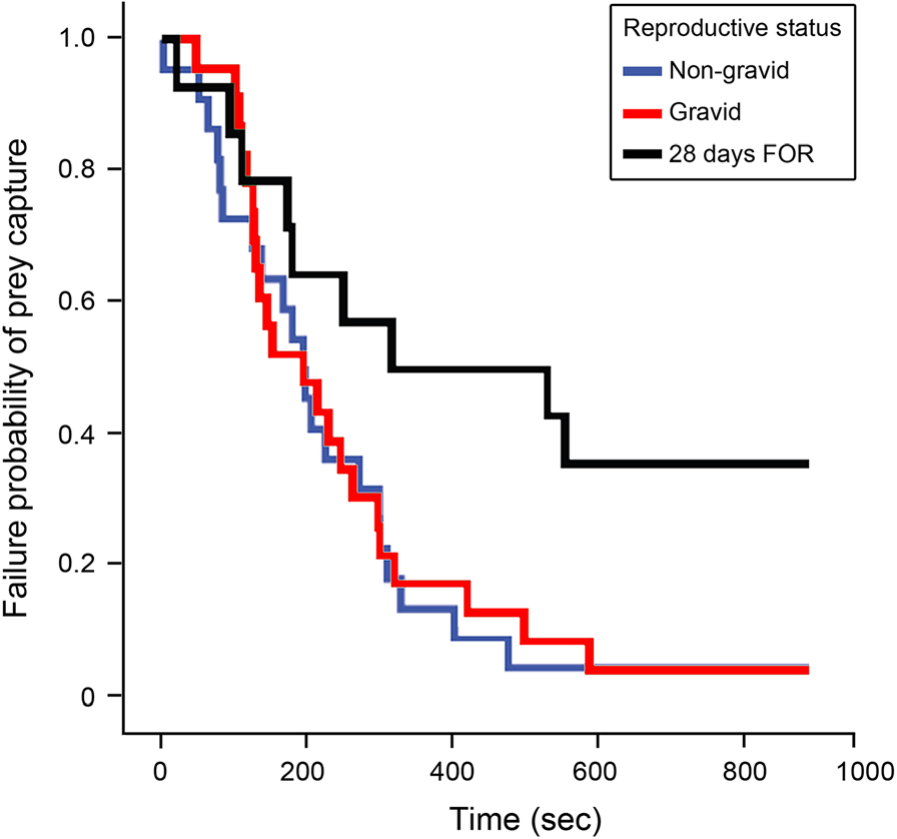
**Kaplan-Meier Failure Time Analysis.** The probability of failure to capture prey over time (trial duration = 900 seconds) among non-gravid, gravid, and female *Centruroides sculpturatus* 28 days following offspring removal (FOR).

**Figure 2:**
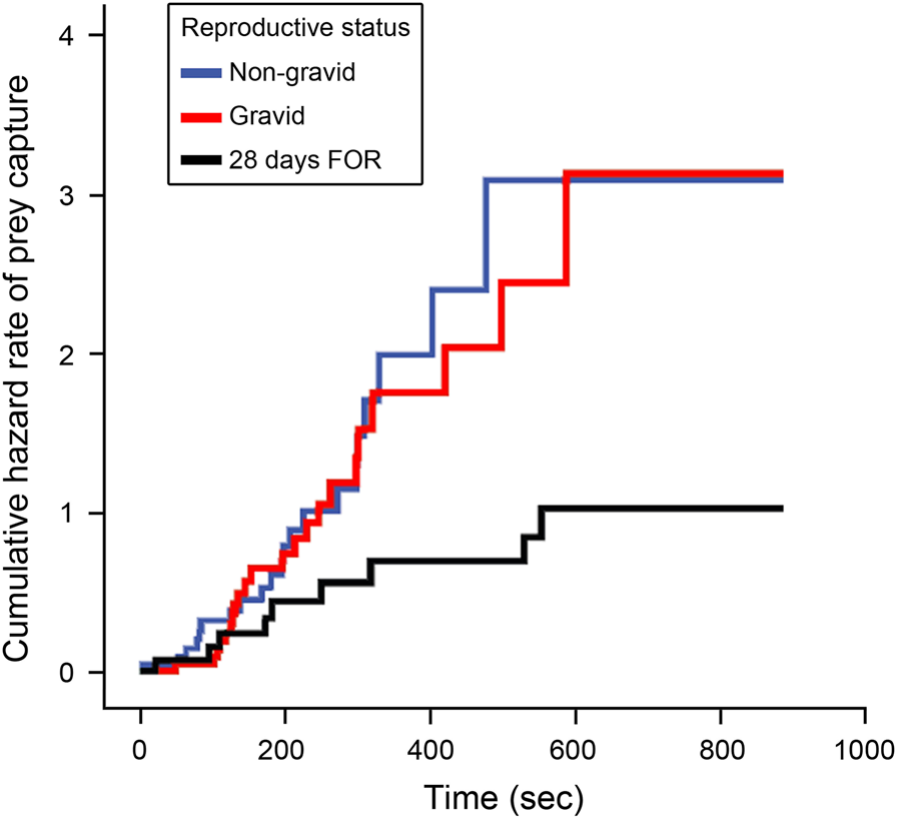
**Cox-Proportional Hazards Model.** The cumulative hazard rate of prey capture over time (trial duration = 900 seconds) among non-gravid, gravid, and female *Centruroides sculpturatus* 28 days following offspring removal (FOR).

The Kaplan-Meier Failure Time Analysis revealed that non-gravid and gravid females captured prey with similar speed (Mantel-Cox test, *X*^*2*^ = 0.092, *df* = 1, *P* = 0.76; Table 3, Figure 1). In contrast, non-gravid (Mantel-Cox test, *X*^*2*^ = 5.97, *df* = 1, *P* = 0.02) and gravid females (Mantel-Cox test, *X*^*2*^ = 5.15, *df* = 1, *P* = 0.023) caught prey significantly faster than females 28 days FOR. The Cox-Proportional Hazards Model showed that differences in overall body size among non-gravid, gravid, and females 28 days FOR did not significantly affect the prey capture rates of females. However, chela size had a significant effect on prey handling times in the comparison of gravid females and females 28 days FOR (Table 2). Gravid female *C. sculpturatus* had significantly wider chelae than females 28 days FOR (gravid females: mean ± SD = 1.70 ± 0.14 mm, n = 23; females 28 days FOR: 1.60 ± 0.14 mm, n = 14; ANOVA, F = 4.47, *df* = 1, *P* = 0.042). Nevertheless, after controlling for differences in body size, non-gravid and gravid females caught prey faster than females 28 days FOR, and the hazard ratio of prey capture for non-gravid and gravid females was similar (Table 2; Figure 2). Because none of the females exhibiting maternal care (n = 20) or females 24 hours FOR (n = 17) successfully captured prey within the 900-second trial period, prey capture time was right-censored for these groups, and these females were excluded from both the Kaplan-Meier Failure Time Analysis and the Cox-Proportional Hazards Analysis.

**Table 3:** **Mean prey capture time (seconds) of non-gravid and gravid female *Centruroides sculpturatus, *** **and of females exhibiting maternal care and females 24 hours and 28 days following offspring removal**

**Reproductive status**	**n**	**Mean ± SD**	**Range**
		**(seconds)**	**(seconds)**
Non-gravid females	22	231.3 ± 191.8	3– > 900
Gravid females	23	251.1 ± 195.5	48–588
Females exhibiting maternal care	20	-	-
Females 24 hours following offspring removal	17^a^	-	-
Females 28 days following offspring removal	14^b^	480.8 ± 356.2	21– > 900

^a^Three females exhibiting maternal care died less than 24 hours following offspring removal.

^b^Three post-parturient females died less than 28 days following offspring removal.

The foraging strategy used by females differed among reproductive groups (Table 4). Non-gravid females used active foraging and ambush predation with similar frequency, compared to gravid (G = 0.051, *df* = 1, *P* = 0.82) and females 28 days FOR (G = 1.88, *df* = 1, *P* = 0.17). Gravid females also used active and ambush foraging with similar frequency, compared to females 28 days FOR (G = 1.26, *df* = 1, *P* = 0.26). However, females exhibiting maternal care used active foraging more frequently than non-gravid (G = 11.37, *df* = 1, *P* = 0.001), gravid (G = 9.72, *df* = 1, *P* = 0.002), and females 28 days FOR (G = 4.39, *df* = 1, *P* = 0.036). Trials examining the foraging behavior of females 24 hours FOR were excluded from this analysis due to low sample size.

**Table 4:** **Frequency of foraging strategies used by non-gravid and gravid female *Centruroides sculpturatus*, and by females exhibiting maternal care and females 28 days following offspring removal**

**Reproductive status**	**Frequency of foraging strategy**	
	**Active**	**Ambush**
	**searching (%)**	**predation (%)**
Non-gravid females	7 (38.8%)	11 (61.2%)
Gravid females	6 (42.9%)	8 (57.1%)
Females exhibiting maternal care	8 (100%)	0 (0%)
Females 28 days following offspring removal	6 (66.7%)	3 (33.3%)

Global Likelihood Ratio, G = 12.8, *df* = 3, *P* = 0.005.

## Discussion

For viviparous females, activities associated with reproduction may adversely affect resource acquisition. Increases in body mass due to developing offspring and nutrient allocation to offspring during gestation may hinder locomotion in gravid females [6,14]. These reproductive costs have the potential to hamper the prey capture abilities of these females [29]. Contrary to our expectations, the predatory efficiency of gravid female *C. sculpturatus* did not decrease as a direct consequence of their reproductive status. This pattern suggests that for gravid Arizona Bark Scorpions, significant increases in body mass do not noticeably inhibit movements as they pertain to pursuing and subduing prey, or significantly deplete the energy necessary for prey capture.

In species that exhibit maternal care, reproductive costs may extend beyond gestation and manifest themselves during and subsequent to the brooding period [11,30]. In this study, none of the females exhibiting maternal care or females 24 hours FOR successfully captured prey within the 900-second trial period. The majority of these trials were characterized by failed grasping attempts, or by the inability of females to maintain a hold on the prey after capturing it with their chelae. These findings illustrate that reproductive costs in terms of decreased predatory efficiency are highest for female *C. sculpturatus* after parturition, and that these females do not immediately recover from the costs associated with giving birth and providing maternal care. What factors may contribute to the decrease in prey capture ability in brooding Arizona Bark Scorpions? Following birth, offspring move from inside the female’s abdomen to her back. This redistribution of offspring mass may impede a brooding female’s ability to move efficiently, thus increasing the difficulty of prey capture. Further, the offspring of scorpions are mobile following birth, and often cling to the legs, underside, and tails of their mothers (M. M. Webber, personal observations), which may increase the difficulty in locating or pursuing a prey item, as well as compromise the stinging mechanics of brooding females, which may increase the time needed to subdue prey. Although brooding females and females 24 hours FOR exhibited a marked decrease in prey capture abilities, female *C. sculpturatus* in the wild may reduce the negative impacts of maternal care on foraging success by pursuing smaller prey or different prey types that may be easier to subdue.

The inability of brooding females and females 24 hours FOR to capture prey suggests that physiological costs associated with maternal care are responsible for the decrease in the predatory efficiency of these scorpions. Alternatively, the failure of brooding females and females 24 hours FOR to catch prey could result from a decrease in the motivation to feed during and subsequent to the brooding period. In 10 separate prey-handling trials, post-parturient females exhibiting maternal care did not attempt to capture prey. (As previously stated, these trials were excluded from the statistical analyses of prey capture frequency and time to prey capture.) Reproductive females may experience seasonal anorexia, a period during which females do not feed, despite the fact that prey is available in their habitats [31,32]. Although seasonal anorexia may lead to poor body condition in females subsequent to the reproductive season, this behavior may allow females to compensate for reduced predatory performance by conserving the energy that would be invested in unsuccessful foraging. A reduction in the foraging behavior of female *C. sculpturatus* exhibiting maternal care may also be caused by a behavioral shift in which females refrain from feeding to decrease the chances of offspring injury or mortality resulting from retaliatory behavior of prey. Further, lessened foraging behavior may improve the chances of offspring survival by reducing the probability of filial cannibalism, when females consume their young to replenish energy invested in reproduction [33,34]. Nevertheless, an earlier study showed that filial cannibalism by brooding female scorpions is a rare occurrence [24], and indeed we did not observe this behavior in our study. Still, the hypotheses that post-parturient females conserve energetic resources and display behaviors that may decrease the occurrence of filial cannibalism by exhibiting seasonal anorexia are not mutually exclusive, as both outcomes may increase the survival and reproductive fitness of female *C. sculpturatus* in nature.

Females 28 days FOR caught prey with a higher success rate than females displaying maternal care and females 24 hours FOR, but still took significantly longer to capture prey than non-gravid and gravid females. In addition, females 28 days FOR were significantly less likely to capture prey than non-gravid and gravid females. These findings indicate that reproductive costs in *C. sculpturatus* extend at least 4 weeks beyond the maternal care period. Our experimental design required females 28 days FOR to remain in the trials for 21 days longer than non-gravid and gravid females, which may have resulted in stress-induced changes in feeding behavior. Nonetheless, the observation that females 28 days FOR successfully pursued, captured, and ingested prey suggests that the extended time in the experimental trials had a negligible effect on the foraging success of these scorpions. Because the predatory efficiency of female *C. sculpturatus* is compromised during and after the maternal care period, poor feeding performance following parturition may be at least partly responsible for the relatively long recovery time experienced by reproductive females. It is important to point out that to control for differences in the duration of maternal care, we removed all offspring from females after 5 days. Under natural conditions, female scorpions are known to carry offspring for up to 51 days [21], and hence for female *C. sculpturatus* in nature, the negative effects of reproduction on predatory efficiency may be greater and extend for a longer time period than our trials allowed us to observe. The length of recovery time for female *C. sculpturatus* likely has significant fitness consequences, because female Arizona Bark Scorpions that replenish their energetic resources faster may have increased survival rates, as well as a higher likelihood of engaging in additional reproductive bouts.

Differences in overall body size and chela size were not significant predictors of variation in prey-handling time between non-gravid and gravid females, or between non-gravid females and females 28 days FOR. However, chela size significantly affected prey handling time in the comparison between gravid females and females 28 days FOR. The dimensions of scorpion chelae are strongly correlated with maximum pincer force, and individuals with relatively shorter and wider chelae have a greater mechanical advantage than those with longer, thinner chelae [35]. In this study, gravid female *C. sculpturatus* had significantly wider chelae than females 28 days FOR, which may have conferred the former group an advantage when catching and subduing prey by enabling those individuals to maintain a stronger grasp on prey items, thus decreasing their prey-handling time.

We observed differences in the foraging strategies used by females in different reproductive groups. In the 8 prey handling trials in which the foraging strategy used by brooding females could be determined, those scorpions actively pursued prey more frequently than non-gravid, gravid, and females 28 days FOR. Although none of the females providing maternal care successfully captured prey, all individuals included in our analyses attempted to do so, and actively pursued crickets placed in their enclosures. What factors may cause brooding females to prefer active foraging? Post-parturient females often have depleted energy reserves following the birth of offspring [36], and females that do not replenish those reserves relatively quickly suffer higher rates of mortality [37]. During the maternal care period, females in poor physiological condition may switch from an ambush to an active foraging strategy to increase their frequency of prey encounters. Despite the fact that active foraging may be energetically costly and increase the predation risk for brooding females, the need to quickly replenish energetic resources may offset the potential negative impacts of this foraging strategy. On the other hand, why may non-gravid, gravid, and females 28 days FOR utilize an ambush strategy more frequently than brooding females? Females in those three groups were more successful at catching prey and may be in better physiological condition than brooding females, and thus may rely on their energy reserves during periods of low food abundance. Further, for these females the increased energy expenditure and predation risk associated with active searching may outweigh the advantage of increased prey encounter rates associated with wide foraging.

## Conclusion

Life history theory suggests that tradeoffs exist in the resources allocated between current and future reproductive episodes, and that the outcome of reproductive investments is dictated by the current physiological state of an organism. We predicted that reproductive costs negatively influence predatory efficiency in both gravid and brooding female *C. sculpturatus*. Contrary to our expectations, gravid females did not exhibit a decrease in their predatory abilities. However, maternal care given to current offspring by female *C. sculpturatus* did lead to a significant reduction in the females’ ability to capture prey. Further, even 28 days FOR, females were still not as efficient at capturing prey as non-gravid and gravid females. Our results demonstrate that the magnitude of reproductive tradeoffs fluctuates over the course of a reproductive bout, and emphasize the importance of isolating stage-specific costs when investigating reproductive tradeoffs. We also observed changes in the foraging strategy of brooding females, as they used an active foraging strategy more frequently than non-gravid, gravid, and females 28 days FOR. This alteration in foraging behavior may increase the likelihood of encountering prey, but may also negatively impact opportunities for future reproduction by increasing the susceptibility of these females to predation.

Our invertebrate model may provide a framework on which to base additional hypotheses regarding physiological, ecological, and evolutionary tradeoffs involving reproduction. For example, females that are in poor body condition may decrease their current reproductive investment by altering the number or size of offspring produced [38,39], or reduce the duration of maternal care given to offspring [40]. Future studies investigating the influence of resource availability and body condition on the reproductive investments (i.e. reproductive frequency, offspring number, and offspring size) and survival of female Arizona Bark Scorpions may uncover additional tradeoffs experienced by reproductive females. Finally, our findings regarding antagonistic interactions between feeding and reproduction in female *C. sculpturatus* illustrate the pervasive nature of reproductive costs for viviparous females, and help elucidate how these costs may influence the diversity of reproductive strategies observed in nature.

## Competing interests

The authors declare that they have no competing interests.

## Authors’ contributions

MMW conceived the study. MMW and JAR-R planned the experimental design. MMW conducted the experimental trials and carried out data analysis. JAR-R assisted with data interpretation. MMW and JAR-R wrote the manuscript. Both authors read and approved the final manuscript.
